# Adduct of Malondialdehyde to Hemoglobin: A New Marker of Oxidative Stress That Is Associated with Significant Morbidity in Preterm Infants

**DOI:** 10.1155/2013/901253

**Published:** 2013-04-28

**Authors:** Cécile Cipierre, Stéphane Haÿs, Delphine Maucort-Boulch, Jean-Paul Steghens, Jean-Charles Picaud

**Affiliations:** ^1^Département de Néonatologie, CHU Angers, 49100 Angers, France; ^2^Département de Néonatologie, Hôpital de la Croix Rousse, Hospices Civils de Lyon, 69004 Lyon, France; ^3^Centre de Recherche en Nutrition Humaine Rhône-Alpes, Centre hospitalier Lyon-Sud, 69310 Pierrre Bénite, France; ^4^Département de Biostatistique, Hospices Civils de Lyon, 69003 Lyon, France; ^5^Université Claude Bernard Lyon I, 69100 Villeurbanne, France; ^6^CNRS UMR5558, Laboratoire de Biométrie et Biologie Evolutive, Equipe Biostatistique Santé, 69310 Pierre-Bénite, France; ^7^Centre de Biologie Sud, UF Nutrition et Métabolisme, Centre hospitalier Lyon-Sud, Hospices Civils de Lyon, 69310 Pierrre Bénite, France

## Abstract

Preterm infants (PT) are particularly exposed to oxidative stress (OS), and a blood-sparing marker, the malondialdehyde adduct to hemoglobin (MDA-Hb), may be useful to accurately assess OS-related neonatal morbidity. In a prospective study, MDA-Hb concentrations were assessed in two groups of PT, one with and one without severe neonatal morbidity as estimated by a composite index of severe morbidity (ISM). All PT born in a single tertiary care NICU (<32 weeks and birth weight <1500 g) were consecutively included. MDA-Hb and blood glutathione (GSH) concentrations were measured by liquid chromatography-mass spectrometry during the first 6 weeks of life. Linear regressions and a multilevel model were fitted to study the relationship between MDA-Hb or GSH and ISM. Of the 83 PT (mean ± SD: 28.3 ± 2 weeks, 1089 ± 288 g), 21% presented severe neonatal morbidity. In the multivariate model, MDA-Hb concentrations were significantly higher in the ISM+ group than in the ISM– group during the first 6 weeks of life (*P* = 0.009). No significant difference in GSH concentrations was observed between groups (*P* = 0.180). MDA-Hb is a marker of interest for estimating oxidative stress in PT and could be useful to evaluate the impact of strategies to improve perinatal outcomes.

## 1. Introduction

As very low birth weight (VLBW) infants present an imbalance between the prooxidant and antioxidant systems [1–3], they are at high risk for oxidative-stress- (OS-) related damage. Antioxidant enzymes mature in late gestation, and the maternal-fetal transfer of antioxidant molecules like alpha-tocopherol and ascorbic acid is not complete in premature neonates [4]. Yet, these infants also often require oxygen therapy and a parenteral nutrition. Although parenteral nutrition solutions contain antioxidant molecules like vitamins A, C, and E, they also contain polyunsaturated fatty acids that are particularly sensitive to peroxidation, which generates toxic byproducts of the reactive oxygen species [5, 6]. The lipid peroxidation in these solutions depends on their composition and is increased by light exposure [7–9]. A relationship has been suggested between lipid peroxidation and several common morbidities of prematurity, including bronchopulmonary dysplasia (BPD), retinopathy of prematurity, periventricular leukomalacia, and necrotizing enterocolitis (NEC) [10–13]. However, the studies show discrepancies because of differences in peroxidation assessment. Malondialdehyde (MDA) is the most studied product of polyunsaturated fatty acid peroxidation, but most assays have been developed on the basis of its derivatization with thiobarbituric acid (TBA), which has poor specificity [14, 15]. Other methods, such as liquid chromatography coupled with mass spectrometry, have been proposed to improve MDA assessment [16, 17]. We recently validated a new sensitive and specific method to measure MDA adduct to hemoglobin (MDA-Hb) in neonates [18]. This method would facilitate OS evaluation over several weeks or months based on the stability of MDA-Hb, as its elimination depends on the lifespan of the erythrocyte [19]. As MDA-Hb is measured in erythrocytes, additional blood despoliation is avoided, which makes the method well suited to VLBW infants. 

This pilot study sought to determine the relationship between blood MDA-Hb concentrations and neonatal morbidity in VLBW infants. A secondary objective was to assess the influence of the perinatal condition on MDA-Hb concentrations.

## 2. Methods

### 2.1. Population

All PT born at a gestational age (GA) of 24 to 31 weeks, with a birth weight (BW) below 1500 g, and admitted before the first 6 hours of life to our tertiary care neonatal unit at Croix Rousse University Hospital, Lyon, France, were consecutively enrolled in this prospective study. Infants with major congenital abnormalities (including cardiac, neurological, renal, and digestive malformation) or metabolic disease, and those requiring surgery, were excluded. The study was totally integrated into the usual care of PT hospitalized in the unit. No additional blood sampling was needed for the present study, and the protocols of respiratory and nutritional care did not change during the study. The international recommendations for nutrition were followed for all infants. All parents signed an informed consent form. The study was approved by the ethics committee of the University Hospital Center of Lyon, France (*CPP Lyon Sud Est IV*).

### 2.2. Study Design

The patients were included at birth and the study lasted for the first 6 weeks of life or infant transfer/death. The clinical data were collected by a single person (C. Cipierre). We prospectively recorded variables concerning the antenatal context in four categories: fetal growth restriction (FGR) (estimated fetal weight <10th percentile), preeclampsia (characterized by hypertension and proteinuria), suspicion of maternal antenatal infection (positive blood culture, maternal fever ≥ 38.5°C, and C-reactive protein > 20 mg/L), and other causes (metrorrhagia and placental abnormalities). We also collected data at birth: GA, BW, Apgar score at 5 min, and the need for specific intensive resuscitation (intubation, chest compression, or drugs for resuscitation). The infants were considered as growth restricted when BW was <−2 SD [20]. We also collected data concerning respiratory care (assisted ventilation, oxygen supply, and surfactant administration), hemodynamics (vasoactive amine, ibuprofen administration), nutrition (total parenteral supply), and complications during hospitalization (early onset sepsis, late onset sepsis, BPD, NEC, intraventricular haemorrhage, and periventricular leukomalacia). 

A composite index of severe morbidity (ISM) was considered as present (ISM+) within the first 6 weeks of life when the patients presented severe respiratory, neurological, or digestive morbidity. The composite index of severe morbidity (ISM) was considered when at least one of the three morbidities was present. Severe respiratory morbidity was defined by the presence of at least one of the following: oxygen dependency at 36 weeks postconceptional age or median duration of assisted ventilation >+2 SD compared with the median duration of ventilation in the same population of GA infants hospitalized in the service between 2005 and 2007 (109 infants born at GA < 28 weeks: median = 249 hours (IC 95%: 288–417) and 202 infants born at GA ≥ 28 weeks: median = 321 hours (IC 95%: 57–844)) (personal data). Severe neurological morbidity was defined as the existence of at least one of the following abnormalities: severe intraventricular haemorrhage (grade 3 or 4) or periventricular leukomalacia. Severe digestive morbidity was defined as NEC of grade 3 or 4 [21].

### 2.3. Measurement of Oxidative Stress Markers

MDA-Hb and reduced glutathione (GSH), a key antioxidant, were measured once a week in the first 6 weeks of life and it did not require an additional blood sample to be drawn. We assumed that the erythrocytes remaining from routine blood samples taken for electrolyte determination would be sufficient for MDA-Hb assessment.

At birth, MDA-Hb and GSH concentrations were assessed at the time of admission in the neonatal unit, that is, before the first hour of life for inborn infants and before the first 6 hours of life for outborn infants. 

All samples were stored at −80°C until analysis. These markers were measured by a unique laboratory, as previously described [18]. 

The procedure for measuring MDA-Hb consisted of three steps: isolation of Hb and delipidation for avoiding any artifactual lipid peroxidation, hydrolysis and derivatization of the adduct, and then measurement of the adduct. In brief, after a first centrifugation, RBCs (one volume) were washed two times with four volumes of NaCl (9 g/1000 mL), centrifuged (5 minutes, 1000 G). 150 *μ*L of washed and packed RBCs was resuspended in distilled water (450 *μ*L) and freeze-dried at −80°C for 5 minutes then thawed in hot water (30 seconds under water at 60°C). After a second cycle of freeze drying—thawing, Hb solution was obtained by centrifugation for 4 min at 8000 G; one aliquot was used to measure Hb concentration and another aliquot (200 *μ*L) was rapidly delipidated by mixing with 100 *μ*L Folch reagent (methanol/chloroform, 1 vol./2 vol.) and centrifuged for 5 minutes at 13000 G. The delipidated Hb from the top phase was stored at −80°C until analysis. The original method to derivatize plasmatic MDA with diaminonaphthalene (DAN) was previously described [22]. Preliminary experiments showed that to decrease adsorption of the diazepinium (formed between MDA and DAN) to Hb, derivatization had to be done in saline. The quantification was carried out by LC-MS with a dideurated internal standard; the derivatives of MDA and dideuterated MDA were detected at *m*/*z* 195.2 and 197.2, respectively, as described by Steghens et al. [16]. The adduct of MDA to Hb was expressed in nanomol per gram Hb (nanomol/g Hb).

For GSH assessment, we used whole blood (25 *μ*l) kept into the guarding of the needle at the end of venipuncture. As described in [23], GSH was measured after derivatization with *N-ethylmaleimide *(NEM) to avoid any artifactual production of oxidized glutathione (GSSG) due to protein acid precipitation. The method used for GSH measurement by LC-MS discriminates GSNEM detected at *m/z* = 433.7 with a retention time at 2.9 min and GSSG detected at *m/z* = 614.1 with a retention time at 4.4 min. The results are expressed in micromole per liter (*μ*mol/L) of whole blood.

To consider the possible contribution of blood transfusions, we measured the MDA-Hb and whole blood GSH concentrations in five randomly selected packed red cells used in preterm infants included in our study.

### 2.4. Statistical Analysis

All the collected variables are described in the studied population and within both groups defined by the outcome. Categorical variables are presented as numbers and percentages, and continuous variables are presented as median and extreme values. Percentages were compared using the Fisher exact test, and medians were compared using the Wilcoxon test. Differences were considered significant for *P* values < 5%. We compared the concentrations of MDA-Hb and GSH at birth in the two ISM groups (absent/present) with regard to the four antenatal contexts previously described. Linear regressions were fitted to study the relationship between MDA-Hb and ISM (absent/present) in the first 6 weeks of life. Because the MDA-Hb measures were repeated weekly in each infant, a multilevel model with random effects, or frailties, was used to account for correlations between measures in the same infant due to unobserved factors. MDA-Hb values were log-transformed to normalize their distribution. To study the trend over time of the repeated MDA-Hb measurements, time in weeks was introduced into the model as a continuous variable in addition to ISM. Similar models were fitted for GSH. Analyses were performed using R software (R Development Core Team; R: a language for environmental and statistical computing, Vienna: R Foundation, 2008) and SPSS version 15.0 software (Statistical Product and Service Solutions 15.0; SPSS, Inc, Chicago, IL, USA).

## 3. Results

Between February and July, 2009, 83 VLBW infants were consecutively enrolled in this study, 40% of whom were very immature (GA ≤ 28 wks). Seventeen of them (21%) presented a composite index of severe morbidity (ISM+), and 3 of these infants died. The first one died after 4 days from septic shock. Two others died from severe neurological complications (HIV stage 4, status) after 2 and 10 days of life. 

As expected, the characteristics of infants with severe neonatal morbidity (ISM+) were significantly different from those of infants without morbidity (ISM−) (Table 1). The ISM+ group comprised more immature and sicker children who had assisted ventilation, oxygen supply, and parenteral nutrition for longer periods than in the ISM− group. They also more frequently presented patent ductus arteriosus requiring ibuprofen and late onset sepsis, and they received more blood transfusions: 14 of the 17 infants in the ISM+ group had at least one transfusion, and 10 of these had received more than 2 transfusions. The median postnatal age at the first and second transfusions was, respectively, 7 and 12 days for the ISM+ group and 7 and 13 days for the ISM− group. Antenatal factors did not differ significantly between the ISM+ and ISM− groups.

**Figure 1:**
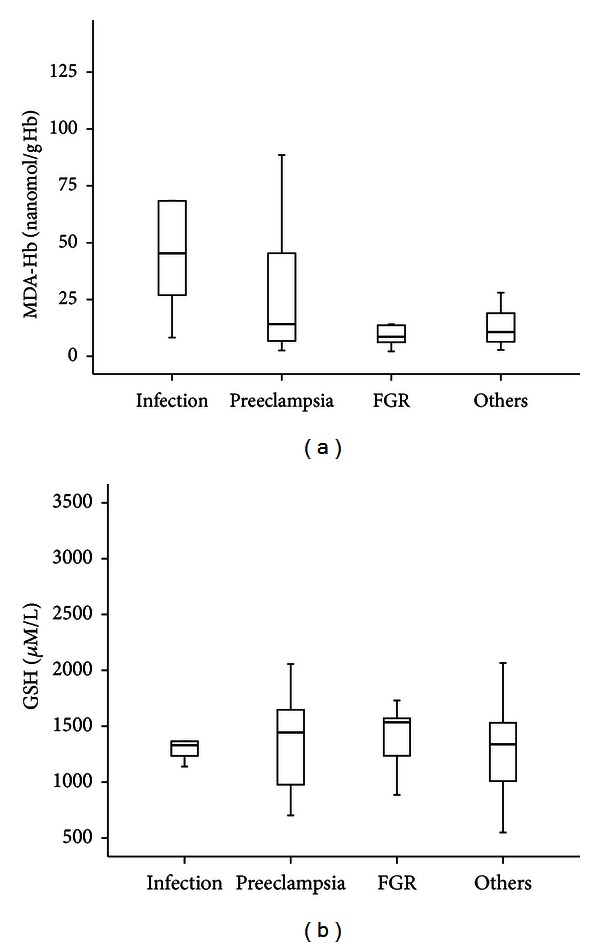
Box plot of the concentrations of malondialdehyde (MDA-Hb) (a) and reduced glutathione (GSH) (b) at birth, in 83 very low birth weight infants, depending on the antenatal context. FGR: fetal growth restriction. Values shown are median levels (25th/75th box; 10th/90th error bars).

MDA-Hb concentrations at birth differed according to the antenatal context (Figure 1). Significantly higher MDA-Hb concentrations were observed in infants with maternal antenatal infection compared with infants without infection (*P* = 0.023). In contrast, GSH concentrations at birth were similar regardless of the prenatal environment (infants with suspicion of maternal antenatal infection compared with infants without, *P* = 0.931).

MDA-Hb concentrations at birth were significantly higher in the ISM+ group than in the ISM− group, but GSH concentrations were similar in the two groups (Table 2). The maximal MDA-Hb value ISM− group was related to only one infant, which can be considered as an outlier. It was a neonate born at 30 weeks by C-section after a 9h ROM in a mother with subnormal PCR (30 mg/L). There was no neonatal infection. 

**Figure 2:**
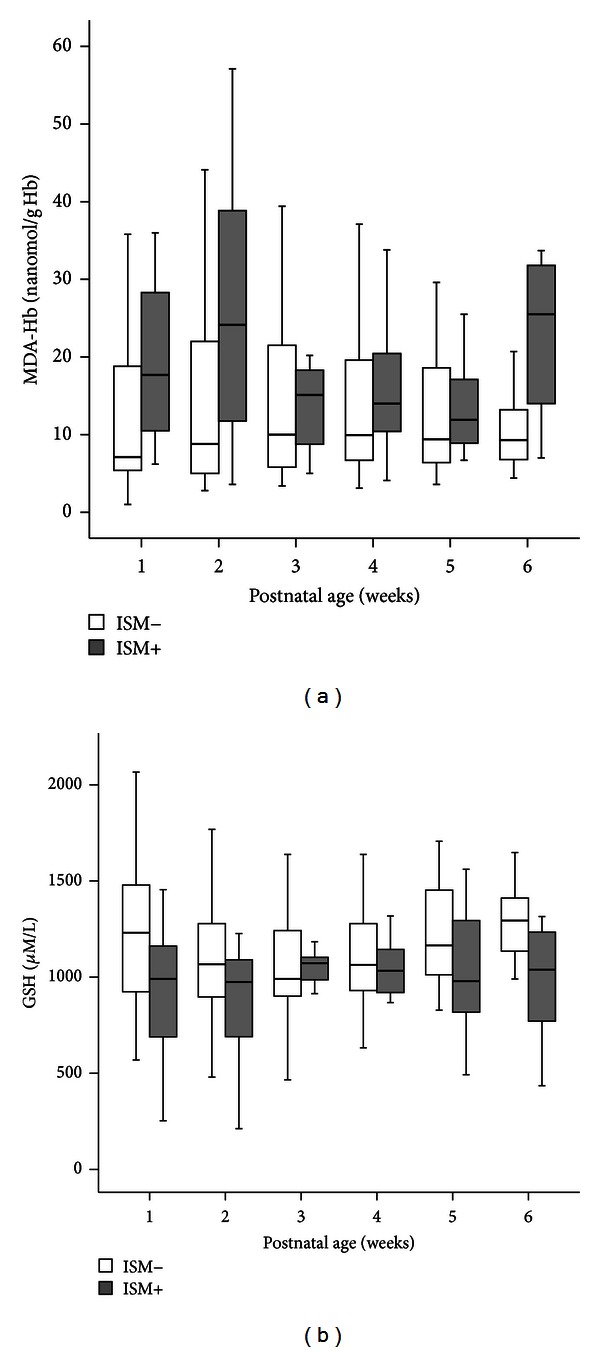
Box plot of the concentrations of malondialdehyde (MDA-Hb) (a) and reduced glutathione (GSH) (b) in the first 6 weeks of life, in 83 very low birth weight infants with (*n* = 17) or without (*n* = 66) a composite index of severe morbidity (ISM). Values shown are median levels (25th/75th box; 10th/90th error bars).

Thereafter, in the first 6 weeks of life, the concentrations of these markers were significantly different between the ISM+ and ISM− groups (Figure 2). Median MDA-Hb concentrations were significantly higher in the ISM+ group during this period (*P* = 0.009), and, at 6 weeks, they were approximately 3-fold higher in ISM+ (25.5 nanomol/g Hb) than in ISM− (9.3 nanomol/g Hb) (*P* = 0.01). Conversely, no significant difference in the GSH concentrations was observed between the groups in the first 6 weeks of life (*P* = 0.18) (Figure 2). 

As the increment of MDA-Hb could be associated to hyperbilirubinemia or phototherapy, we adjusted for presence or absence of phototherapy and for the maximal serum bilirubin concentration, using a multivariate analysis. The difference in MDA-Hb remained statistically significant (resp., *P* = 0.02 and *P* = 0.03). 

The median MDA-Hb and whole blood GSH concentrations in 5 packed red cells were, respectively, 8.4 nanomol/g Hb and 1568 *μ*M/L.

## 4. Discussion

To our knowledge, this is the first report in neonates on the malondialdehyde adduct to hemoglobin (MDA-Hb), a marker of OS measured in red blood cells, that is, without requiring an additional blood sample. We observed that MDA-Hb concentrations were influenced by the clinical context, as described in studies with MDA [11].

Published data about background levels of MDA-Hb in humans are scarce. Kautiainen et al. [24] assessed MDA-Hb concentrations in one healthy adult and in mice. They reported lower values (0.2 and 3.8 nanomol/g Hb) which are not comparable to ours because they used a very different and complex method: using a 4 ml sample volume, the final measurement of the 3 OH Pr Val was obtained after reduction with sodium borohydride (NaBH4), dialysis, and globin precipitation, derivatization with PFPITC, proteolysis with trypsin and pronase, and a last Dowex chromatography. This is not usable in human neonates (blood volume, complex assay). Furthermore, these values were obtained in a very small number of subjects (1 human adult and some mice) and cannot be compared to values obtained in a significant number of newborn infants.

For the purpose of monitoring, Hb can be preferred to DNA because of its better-defined lifespan and more facile chemical identification of adducts. In particular, the estimated half-live of the adduct to N-terminal valine in Hb is about six days [25]; thus, it is possible that this type of adduct may accumulate in the blood of newborn.

The present study has several limitations which need to be addressed. First, this was our definition of the composite index of severe morbidity. The composite index of severe morbidity included several types of neonatal morbidity with different time courses. For example, respiratory morbidity develops over a few weeks, whereas the onset of NEC is quite rapid. Our global approach might be considered as less precise than if we had considered each type of morbidity individually, but a global approach was essential to our study as we aimed to include all the situations carrying significant morbidity. Although the number of patients was not very large, the population was rather homogeneous and representative of the population of PT from 24 to 31 weeks. Moreover, the population was large enough to reveal a relationship between the level of OS and both the antenatal context and the neonatal morbidity. In our population, we found maximal MDA-Hb value in one infant of the control (ISM−) group. It could be considered as an outlier. It could be also a false-positive result. There was no neonatal infection. We do not have any explanation for that high value. However, as we aimed to evaluate whether MDA-Hb could be used as a marker of oxidative stress in a population of preterm infants, we could not expect a 100% specificity.

Our population represented the four classes of etiology in preterm delivery: maternal antenatal infection, preeclampsia, fetal growth restriction (FGR), and other causes (metrorrhagia, placental abnormalities). The first three are associated with inflammation, which is known to affect redox balance [26]. We found the significantly highest MDA-Hb concentration at birth in the cases of maternal antenatal infection, which is characterized by the release of inflammatory cytokines by decidua and fetal membranes, leading to preterm labor and the generation of free radicals and ROS in fetal and maternal circulation [27]. In contrast, GSH concentrations at birth were similar regardless of the prenatal environment and similar to GSH concentrations in healthy adults [23]. Lower GSH concentrations were observed in VLBW infants compared with full-term infants [28], but GSH at birth has not been studied in VLBW infants in relation to the perinatal context, to our knowledge.

Various fundamental and clinical data have underlined the central role of OS in the physiopathology of current neonatal diseases related to prematurity [29–33]. Therefore, higher concentrations of oxidant markers were expected in the sicker infants, and we indeed found an association between MDA-Hb, an index of lipid peroxidation, and neonatal morbidity. This finding agrees with previous findings using other oxidative markers [1, 29, 30, 34]. 

The MDA-Hb concentration at birth could be useful in identifying neonates at high risk for severe morbidity, as we found higher values in the ISM+ group than in the ISM− group. This fits quite well with the results recently published by Perrone et al. [35], reporting significantly higher levels of oxidant markers in the cord blood of preterm infants with NEC than in preterm infants without NEC [29, 34–36]. Higher MDA-Hb concentrations were found in the ISM+ group not only during the first postnatal week, as often described [29, 34, 37], but also over the first 6 weeks of life. 

The evidence of OS persisting for at least a month strongly suggests a long-lasting imbalance between antioxidant and oxidant-generating systems, which causes oxidative damage in preterm infants. 

Depletion of whole blood GSH is known to occur in preterm infants, and GSH correlates with gestational age [38, 39]. Therefore, the GSH concentrations should have been lower in the less mature infants (ISM+ group) than in the ISM− group. This was not the case, suggesting the absence of a clear relationship between GSH concentrations and severe neonatal morbidity. Two explanations for our finding may be the small size of our study population and the influence of blood transfusions (more frequent in the ISM+ group). However, one should note that the GSH concentrations in both the ISM+ and ISM− groups were similar to those of healthy adults, as measured by Steghens et al. using the same dosage method [23]. Very few studies have reported reduced glutathione measurement, and most of these studies concerned cord blood samples from neonates not yet exposed to true OS and compared preterm infants with healthy full-term infants. To our knowledge, this study is the first to evaluate the relationship between severe morbidity in preterm infants at birth and over 6 weeks of age and the GSH concentration. 

In the studies performed several days after birth, lower GSH concentrations (in erythrocytes and cells from tracheal aspirates) were observed in preterm infants with respiratory distress syndrome [39, 40]. In the present study, no difference in GSH concentrations in the ISM+ and ISM− groups was observed in the first 6 weeks of life. The lowest concentration of GSH in the ISM+ group was observed at the time of the MDA-Hb peak, during the second week of life. This could be explained by an abnormality in the antioxidant system in the ISM+ infants, who were exposed to higher levels of OS related to excessive consumption and/or reduced capacities of GSH synthesis due to hepatic immaturity or a deficit in cysteine, the main acid amine regulator of GSH synthesis [41–43].

The infants in the ISM+ group had more blood transfusions, which may have had an impact on the MDA-Hb and GSH concentrations. For example, the MDA-Hb concentrations may have been underestimated due to the influence of blood transfusions. This would explain the MDA-Hb decrease in the third week of life in the ISM+ group. Moreover, the cumulative effect of MDA-Hb over time may also have been affected. On the other hand, the GSH concentrations may have been overestimated in this group, but no conclusions can be drawn regarding our GSH results, which did not differentiate the groups but nevertheless remain interesting from a clinical point of view. To estimate the impact of transfusions, we had to measure the MDA and GSH concentrations in five randomly selected packed red cells used in preterm infants included in our study. Both MDA-Hb and whole blood GSH concentrations in packed red cells were neither very low nor very high. As they were close to those measured in healthy full-term neonates at birth [18], the contribution of blood transfusions to MDA-Hb and GSH levels in preterm infants is unlikely.

It appears that the OS level in the ISM+ group was such that oxygen-free radical production, as reflected by the MDA adduct to hemoglobin, exceeded the antioxidant defense system. The evidence of a strong pro/antioxidant imbalance raises many questions about the involvement of OS in the physiopathology of neonatal complications, without being able to establish a causal link. 

In conclusion, the present study is the first step in the validating technique to evaluate postnatal OS without the need of additional blood sampling. The higher MDA-Hb concentrations in the sicker infants suggest that MDA-Hb is a marker of interest for estimating and quantifying OS in VLBW infants. This noninvasive method may help to enhance our understanding of OS, thereby improving the accuracy of modifications in therapeutics (assisted ventilation, oxygen therapy, and parenteral nutrition) to reduce free radical generation and providing guidelines for developing future applications of antioxidant therapy in premature infants. Although these findings require confirmation in a larger population, the results of our study provide insight into the relationship between MDA-Hb and neonatal morbidity.

## Figures and Tables

**Table 1:** Characteristics of 83 very preterm infants with (ISM+) or without (ISM−) composite index of severe morbidity (ISM).

	All *N* = 83	ISM+ n = 17	ISM− n = 66	P value*
Singleton, *n* (%)	62 (74.7)	14 (82.3)	48 (72.7)	0.07
Antenatal steroids, *n* (%)	74 (89.1)	14 (88.3)	60 (90.9)	0.38
Fetal growth restriction, *n* (%)	11 (13.3)	1 (5.9)	10 (15.2)	0.45
Preeclampsia, *n* (%)	10 (12)	0 (0)	10 (15.2)	0.11
Maternal antenatal infection, *n* (%)	5 (6.0)	1 (5.9)	4 (6.1)	1.00
Others causes, *n* (%)	57 (68.7)	15 (88.2)	42 (63.6)	0.08
Cesarean section, *n* (%)	50 (62.6)	7 (41.2)	45 (68.2)	0.05
Gestational age at birth, weeks	28.7 (24.0–31.6)	26.0 (24.0–30.4)	29.0 (24.5–31.6)	<0.01
Birth weight, grams	1085 (570–1500)	765 (570–1500)	1120 (690–1500)	<0.01
Male, *n* (%)	39 (47.0)	11 (64.7)	28 (44.4)	0.10
Apgar score at 5 min, value	9 (2–10)	8 (6–10)	9 (2–10)	0.09
Resuscitation in DR, *n* (%)	49 (59.0)	14 (82.4)	35 (53.0)	0.08
RDS, *n* (%)	80 (96.4)	17 (100.0)	63 (95.5)	0.37
Surfactant, *n* (%)	51 (61.4)	14 (82.3)	37 (56.0)	0.03
Oxygen therapy, hours	37 (1–1661)	276 (1–1661)	68 (1–948)	<0.01
Assisted ventilation, hours	64 (1–1107)	427 (5–1107)	27 (1–764)	<0.01
Early onset sepsis, *n* (%)	6 (7.2)	4 (23.5)	2 (3.0)	0.01
Late onset sepsis, *n* (%)	22 (26.5)	10 (58.8)	12 (18.2)	<0.01
Persistent ductus arteriosus, *n* (%)	22 (26.5)	8 (47.0)	14 (21.2)	0.03
Blood transfusions, *n* (%)	34 (41.0)	14 (82.3)	20 (30.3)	<0.01
Parenteral nutrition, *n* (%)	63 (75.9)	15 (88.2)	48 (72.7)	<0.01
Parenteral nutrition duration, d	8 (1–35)	13 (1–35)	9 (1–28)	<0.01

Categorical variables are presented as number (%) and continuous variables as median (min–max).

*Comparison between the 2 groups (ISM+ versus ISM−, Mann-Whitney or Chi2 test).

**Table 2:** Concentrations of malondialdehyde (MDA-Hb) and reduced glutathione (GSH) at birth in 83 very preterm infants with (ISM+) or without (ISM−) composite index of severe morbidity (ISM).

	All *N* = 83	ISM+ n = 17	ISM− n = 66	*P* value*
MDA-Hb (nanomol/g Hb)	11.3 (2.2–134.3)	19.9 (6.6–117.5)	9.3 (2.2–134.3)	0.03
GSH (*μ*M/L)	1310 (301–2168)	1295 (301–2168)	1324 (989–2042)	0.50

*Comparison between the 2 groups (ISM+ versus ISM−, Mann-Whitney or Chi2 test).

## Abbreviations

BPD: Bronchopulmonary dysplasia
BW: Birth weight
ISM: Index of severe morbidity
DAN: Diaminonaphthalene
FGR: Fetal growth restriction
GA: Gestational age
GSH: Reduced Glutathione
Hb: Hemoglobin
LC-MS: Liquid chromatography-mass spectrometry
MDA: Malondialdehyde
MDA-Hb: Malondialdehyde adduct to hemoglobin
NEC: Necrotizing enterocolitis
OS: Oxidative stress
PT: Preterm infants
RBC: Red blood cell
ROS: Reactive oxygen species
VLBW: Very low birth weight.

## References

[1] Pitkanen OM, Hallman M, Andersson SM (1990). Correlation of free oxygen radical-induced lipid peroxidation with outcome in very low birth weight infants. *Journal of Pediatrics*.

[2] Lindeman JHN, van Zoeren-Grobben D, Schrijver J, Speek AJ, Poorthuis BJHM, Berger HM (1989). The total free radical trapping ability of cord blood plasma in preterm and term babies. *Pediatric Research*.

[3] Sullivan JL, Newton RB (1988). Serum antioxidant activity in neonates. *Archives of Disease in Childhood*.

[4] Robles R, Palomino N, Robles A (2001). Oxidative stress in the neonate. *Early Human Development*.

[5] Helbock HJ, Motchnik PA, Ames BN (1993). Toxic hydroperoxides in intravenous lipid emulsions used in preterm infants. *Pediatrics*.

[6] Pironi L, Guidetti M, Zolezzi C (2003). Peroxidation potential of lipid emulsions after compounding in all-in-one solutions. *Nutrition*.

[7] Picaud JC, Steghens JP, Auxenfans C, Barbieux A, Laborie S, Claris O (2004). Lipid peroxidation assessment by malondialdehyde measurement in parenteral nutrition solutions for newborn infants: a pilot study. *Acta Paediatrica*.

[8] Laborie S, Lavoie JC, Chessex P (2000). Increased urinary peroxides in newborn infants receiving parenteral nutrition exposed to light. *Journal of Pediatrics*.

[9] Jalabert A, Grand A, Steghens JP, Barbotte E, Pigue C, Picaud JC (2011). Lipid peroxidation in all-in-one admixtures for preterm neonates: impact of amount of lipid, type of lipid emulsion and delivery condition. *Acta Paediatrica*.

[10] Saugstad OD (1997). Bronchopulmonary dysplasia and oxidative stress: are we closer to an understanding of the pathogenesis of BPD?. *Acta Paediatrica*.

[11] Inder TE, Darlow BA, Sluis KB (1996). The correlation of elevated levels of an index of lipid peroxidation (MDA-TBA) with adverse outcome in the very low birthweight infant. *Acta Paediatrica*.

[12] Kelly FJ (1993). Free radical disorders of preterm infants. *British Medical Bulletin*.

[13] Thibeault DW (2000). The precarious antioxidant defenses of the preterm infant. *American Journal of Perinatology*.

[14] Del Rio D, Stewart AJ, Pellegrini N (2005). A review of recent studies on malondialdehyde as toxic molecule and biological marker of oxidative stress. *Nutrition, Metabolism and Cardiovascular Diseases*.

[15] Benzie IFF (1996). Lipid peroxidation: a review of causes, consequences, measurement and dietary influences. *International Journal of Food Sciences and Nutrition*.

[16] Steghens JP, Arab K, Rossary A, Soulère L (2006). Conjugated linoleic acid, unlike other unsaturated fatty acids, strongly induces glutathione synthesis without any lipoperoxidation. *British Journal of Nutrition*.

[17] Grand A, Jalabert A, Mercier G (2011). Influence of vitamins, trace-elements and iron on lipid peroxidation reactions in all-in-one admixtures for parenteral nutrition in neonates. *Journal of Parenteral and Enteral Nutrition*.

[18] Cipierre C, Haÿs S, Maucort-Boulch D, Steghens J-P, Picaud J-C Malondialdehyde adducts to hemoglobin: a new marker of oxidative stress suitable for full-term and preterm neonates.

[19] Tornqvist M, Kautiainen A (1993). Adducted proteins for identification of endogenous electrophiles. *Environmental Health Perspectives*.

[20] Usher R, McLean F (1969). Intrauterine growth of live-born Caucasian infants at sea level: standards obtained from measurements in 7 dimensions of infants born between 25 and 44 weeks. *The Journal of Pediatrics*.

[21] Walsh MC, Kliegman RM (1986). Necrotizing enterocolitis: treatment based on staging criteria. *Pediatric Clinics of North America*.

[22] Steghens JP, Van Kappel AL, Denis I, Collombel C (2001). Diaminonaphtalene, a new highly specific reagent for HPLC-UV measurement of total and free malondialdehyde in human plasma or serum. *Free Radical Biology and Medicine*.

[23] Steghens JP, Flourié F, Arab K, Collombel C (2003). Fast liquid chromatography-mass spectrometry glutathione measurement in whole blood: micromolar GSSG is a sample preparation artifact. *Journal of Chromatography B*.

[24] Kautiainen A, Tornqvist M, Svensson K, Osterman-Golkar S (1989). Adducts of malonaldehyde and a few other aldehydes to hemoglobin. *Carcinogenesis*.

[25] Kautiainen A, Vaca CE, Granath F (1993). Studies on the relationship between hemoglobin and DNA adducts of malonaldehyde and their stability in vivo. *Carcinogenesis*.

[26] Valko M, Leibfritz D, Moncol J, Cronin MTD, Mazur M, Telser J (2007). Free radicals and antioxidants in normal physiological functions and human disease. *International Journal of Biochemistry and Cell Biology*.

[27] Woods JR (2001). Reactive oxygen species and preterm premature rupture of membranes—a review. *Placenta*.

[28] Küster A, Tea I, Ferchaud-Roucher V (2011). Cord blood glutathione depletion in preterm infants: correlation with maternal cysteine depletion. *PLoS ONE*.

[29] Inder TE, Graham P, Sanderson PGK, Taylor BJ (1994). Lipid peroxidation as a measure of oxygen free radical damage in the very low birthweight infant. *Archives of Disease in Childhood*.

[30] Varsila E, Pitkanen O, Hallman M, Andersson S (1994). Immaturity-dependent free radical activity in premature infants. *Pediatric Research*.

[31] Weinberger B, Nisar S, Anwar M, Ostfeld B, Hegyi T (2006). Lipid peroxidation in cord blood and neonatal outcome. *Pediatrics International*.

[32] Vento M, Moro M, Escrig R (2009). Preterm resuscitation with low oxygen causes less oxidative stress, inflammation, and chronic lung disease. *Pediatrics*.

[33] O'Donovan DJ, Fernandes CJ (2004). Free radicals and diseases in premature infants. *Antioxidants and Redox Signaling*.

[34] Buonocore G, Perrone S, Longini M (2002). Oxidative stress in preterm neonates at birth and on the seventh day of life. *Pediatric Research*.

[35] Perrone S, Tataranno ML, Negro S (2012). May oxidative stress biomarkers in cord blood predict the occurrence of necrotizing enterocolitis in preterm infants?. *Journal of Maternal-Fetal and Neonatal Medicine*.

[36] Perrone S, Tataranno ML, Negro S (2010). Early identification of the risk for free radical-related diseases in preterm newborns. *Early Human Development*.

[37] Buonocore G, Perrone S, Longini M, Terzuoli L, Bracci R (2000). Total hydroperoxide and advanced oxidation protein products in preterm hypoxic babies. *Pediatric Research*.

[38] Jain A, Mehta T, Auld PA (1995). Glutathione metabolism in newborns: evidence for glutathione deficiency in plasma, bronchoalveolar lavage fluid, and lymphocytes in prematures. *Pediatric Pulmonology*.

[39] Németh I, Boda D (1994). Blood glutathione redox ratio as a parameter of oxidative stress in premature infants with IRDS. *Free Radical Biology and Medicine*.

[40] Lavoie JC, Chessex P (1997). Gender and maturation affect glutathione status in human neonatal tissues. *Free Radical Biology and Medicine*.

[41] Vina J, Vento M, Garcia-Sala F (1995). L-cysteine and glutathione metabolism are impaired in premature infants due to cystathionase deficiency. *American Journal of Clinical Nutrition*.

[42] Wu G, Fang YZ, Yang S, Lupton JR, Turner ND (2004). Glutathione metabolism and its implications for health. *Journal of Nutrition*.

[43] Lu SC (1999). Regulation of hepatic glutathione synthesis: current concepts and controversies. *FASEB Journal*.

